# Killing Two Angry Birds with One Stone: Autophagy Activation by Inhibiting Calpains in Neurodegenerative Diseases and Beyond

**DOI:** 10.1155/2019/4741252

**Published:** 2019-02-14

**Authors:** Jonasz Jeremiasz Weber, Priscila Pereira Sena, Elisabeth Singer, Huu Phuc Nguyen

**Affiliations:** ^1^Institute of Medical Genetics and Applied Genomics, University of Tübingen, Calwerstraße 7, 72076 Tübingen, Germany; ^2^Department of Human Genetics, Ruhr-University Bochum, Universitätsstraße 150, 44801 Bochum, Germany

## Abstract

Proteolytic machineries execute vital cellular functions and their disturbances are implicated in diverse medical conditions, including neurodegenerative diseases. Interestingly, calpains, a class of Ca^2+^-dependent regulatory proteases, can modulate the degradational system of autophagy by cleaving proteins involved in this pathway. Moreover, both machineries are common players in many molecular pathomechanisms and have been targeted individually or together, as a therapeutic strategy in experimental setups. In this review, we briefly introduce calpains and autophagy, with their roles in health and disease, and focus on their direct pathologically relevant interplay in neurodegeneration and beyond. The modulation of calpain activity may comprise a promising treatment approach to attenuate the deregulation of these two essential mechanisms.

## 1. Introduction

Proteolytic machineries of eukaryotic cells are key players in the regulation of protein function or the maintenance of cell homeostasis. Importantly, they act as modifiers of numerous neurodegenerative proteopathies, including classical medical conditions such as Alzheimer disease (AD), Parkinson disease (PD), and the group of polyglutamine (polyQ) disorders. This link is evident as the nature of these diseases, i.e., the occurrence of structurally abnormal toxic proteins, provokes an overload of these systems, leading to their disruption, loss of cellular integrity, and eventually neuronal demise [1]. Beyond neurodegeneration, proteostatic processes are implicated in further medical conditions like, for instance, cancer, cardiovascular disorders, and diabetes [2–4]. This multifarious involvement emphasizes the value of targeting these machineries therapeutically.

In this review, we focus on two major proteolytic machineries of the cell, the calpain protease system and autophagy, which both have been scrutinized in the context of neurodegenerative disorders and other diseases for the last two decades. As often the case with complex cellular pathways, both proteolytic machineries are strongly interconnected and the deregulation of one of them inevitably leads to repercussion on the other. By shedding new light on the impact of calpains on autophagy and vice versa, we aim to work out points of vantage for therapeutic applications, which only target one but may hit both compromised proteolytic systems. Consequently, future disease-treating approaches may kill those rather angry birds, namely overactivated calpains and impaired autophagy, with only one stone.

## 2. Calpains and Autophagy in Neurodegeneration and Other Medical Conditions

### 2.1. Calpains

#### 2.1.1. Calpain Basics

The regulation of protein structure, function, localization, or lifetime is mediated by a vast range of posttranslational modifications (PTMs). Amongst those, proteolytic processing constitutes a profound mechanism, which spans from the removal of single amino acids to longer peptides or whole domains of the targeted protein. One class of enzymes responsible for this modification is calpains, firstly described as a Ca^2+^-activated neutral proteinase in rat brain [5]. The later-promoted term ‘calpain' is a portmanteau, which consists of the two syllables ‘cal' in reference to Ca^2+^ or Ca^2+^-binding proteins and ‘pain' as an allusion to structurally related cysteine proteases like papain from plants or clostripain from* Clostridium* [6]. Calpain and their homologs can be found in unicellular and multicellular organisms, from animals, over plants, fungi, yeast, and down to bacteria [4].

**Figure 1:**
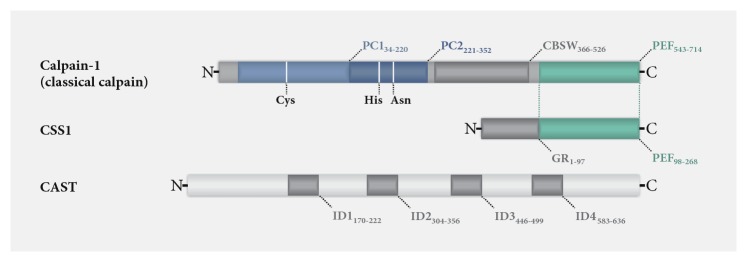
*Domain composition and structure of calpain-1, CSS1, and CAST.* Conventional classical calpains are present as a large protease unit, such as the here depicted calpain-1, and the calpain small subunit 1 (CSS1). Both share a C-terminal Ca^2+^-binding penta-EF-hand (PEF) domain. Calpain-1 further contains an N-terminal proteolytic CysPc domain, consisting of core domains PC1 and PC2, which also bind Ca^2+^ ions. Amino acid positions of the catalytic triad of calpain-1 are indicated by vertical white lines. In addition, a calpain-like *β*-sandwich domain (CBSW) is located between the CysPc and the PEF domain. CSS1 features, moreover, an N-terminal glycine-rich (GR) hydrophobic domain. The endogenous inhibitor calpastatin (CAST) contains four structurally flexible inhibitory domains (ID1-4) of which each can inhibit one calpain molecule. Illustrations of calpain-1, CSS1, and CAST are based on data retrieved from the UniProt database (respective identifiers P07384-1, P04632-1, and P20810-1).

Structurally, all calpains are characterized by their conserved proteolytic domain (CysPc), which is subdivided in the two protease core domains PC1 and PC2. Together with more than 40 different other protein domains or motifs, the CysPc domain forms multiple variants of calpains in a modular principle. The human genome encodes 15 different calpains, divided into two main groups: classical (calpains-1-14) and nonclassical calpains (calpain-5, calpain-6, calpain-7, calpain-10, calpain-15, and calpain-16). Classical calpains feature a C-terminal Ca^2+^-binding penta-EF-hand (PEF) domain. Via this domain, members like calpain-1 and calpain-2, which are referred to as conventional classical calpains, exhibit a vital interaction with the regulatory calpain small subunit 1 (CSS1, formerly known as calpain-4) [7, 8]. Nonclassical calpains lack both the PEF domain and the interaction with a regulatory subunit [4, 8, 9]. The direct antagonist of these proteases is calpastatin (CAST), the only known endogenous, ubiquitously expressed, and highly specific proteinaceous inhibitor of classical calpains. Altogether, calpains, regulatory subunits, and CAST form the intracellular calpain system [10, 11]. A structural representation of calpain-1, CSS1, and CAST is shown in Figure 1. Calpain expression depends largely on the respective isoform: calpain-1, together with the regulatory subunit CSS1, is expressed ubiquitously, and isoforms such as calpain-2, calpain-5, and calpain-10 are found in most cells. However, other calpains, like the skeletal muscle-specific calpain-3, show expression patterns restricted to distinct tissues [8].

The activation mechanism of calpains has been controversially discussed and led to the formulation of different explanatory scenarios [12]. However, X-ray crystallography of Ca^2+^-bound calpain-2 together with CSS1 and CAST shed light on the precise mechanism: in a fully activated state, calpain-2-CSS1 heterodimer binds ten Ca^2+^ ions, of which eight are bound to the two PEF domains, and one Ca^2+^ is bound at each PC domain. The Ca^2+^-binding induces structural rearrangements, which then allows the connection of the PC1 and PC2 core domains to a closed active state [13, 14].* In vitro* studies demonstrated that Ca^2+^ concentrations necessary for activation of calpains were in a micro- to millimolar range, which is rather far beyond the nanomolar Ca^2+^ levels in cells under normal physiological conditions. Yet, this apparent contradiction is resolved, as the cellular microenvironment may provide the sufficient Ca^2+^ concentration [12].

Calpains feature a wide range of cellular functions and act in a regulatory way by performing limited proteolysis of substrates, such as enzymes and structural proteins [15, 16]. Their functional involvement ranges from remodeling cytoskeletal elements, regulating cell motility, cell cycle control, and proliferation, via controlling gene expression, inflammation, autophagy, and apoptosis, through to tuning signal transduction and synaptic plasticity in neurons [7, 17–19].

#### 2.1.2. Calpains in Health and Disease

The important role of calpains in a healthy biological system becomes even clearer in the light of the wide-ranging implications of their malfunction in a multitude of human diseases. The deregulation of calpain function and mediation of molecular pathomechanisms by calpains were described in medical conditions such as myopathies, ophthalmic maladies, cardiovascular disorders, cancer, and neurodegeneration.

A whole group of diseases which are based on the direct dysfunctions of calpains was termed* calpainopathies*, comprising a wide spectrum of pathological manifestations [8]. Limb-girdle muscular dystrophy 2A (LGMD2A) was the first-described calpainopathy, which is caused by mutations in the gene encoding muscular calpain-3 (*CAPN3*) [20, 21]. Missense mutations in the calpain-5 gene (*CAPN5*) were associated with an autosomal-dominant form of neovascular inflammatory vitreoretinopathy (ADNIV) [22]. In cardiovascular injuries, mitochondrial calpain-1 was shown to mediate apoptotic effects [23–25]. Moreover, an intriguing association of calpains was made with diabetes, when calpain-10 was identified as a susceptibility gene for type 2 diabetes [26]. Mutations in the skin-specific calpain-12 were shown to worsen the clinical manifestation of autosomal recessive congenital ichthyosis [27].

Calpains also play a role in tumorigenesis by diversely acting on cancer cell migration, survival, and death, rendering these proteases a potential therapeutic target in oncology [28]. The proteases were shown to contribute to tumor progression and to exhibit deregulated expression patterns on one hand. On the other, calpains are acting as executioners of apoptotic cancer cell death, activated by anticancer drugs [29]. For instance, calpain-1 and calpain-2 demonstrated protumorigenic roles in HER2^+^ breast cancer models, as conditional deletion or knockout of CSS1, which is crucial for the activity of these conventional calpains, blocked or delayed tumorigenesis [30]. High calpain-2 expression was associated with the adverse clinical outcome of basal-like and triple-negative invasive breast cancer [31]. However, the proapoptotic or antineoplastic activity of capsaicin was found to be based on increased Ca^2+^ levels and, thereby, calpain-1 and calpain-2 activation, in models of human small cell lung cancer [32, 33].

Lastly, calpains are also implicated in neuronal injury, neurodegenerative disorders, and neuronal aging processes [1, 34]. For instance, these proteases execute Wallerian degeneration and mediate degenerative effects in traumatic brain injury [35–37]. A detrimental calpain overactivation has been detected in many neurodegenerative disorders such as AD, amyotrophic lateral sclerosis (ALS), PD, or the group of polyQ disorders [38–41]. Interestingly, calpains were associated with fragmentation of the respective disease proteins, leading to the generation of breakdown products with an increased toxicity compared to the full-length protein. This includes *α*-synuclein in PD or transactivation response element DNA-binding protein 43 (TDP-43) in ALS, as well as the polyQ disease proteins huntingtin in Huntington disease (HD) and ataxin-3 in Machado-Joseph disease (MJD). Resulting protein fragments were shown to be more harmful to cells or to readily form disease protein aggregates [42–46]. Consequently, inhibition of cleavage by genetically and pharmacologically targeting calpains or by rendering disease proteins cleavage-resistant ameliorated disease-related molecular and behavioral characteristics in respective models of those diseases [46–51]. Overexpression of CAST in animal models of AD and ALS showed beneficial effects by counteracting the intrinsic calpain overactivation [52–54]. Most recently, a neuronal calpainopathy was identified which is caused by* CAPN1*-null mutations, leading to cerebellar ataxia and limb spasticity [55]. Furthermore, calpain-1 and calpain-2 seem to have opposing roles in neuronal function, mediating synaptic plasticity, and neuroprotection versus neurodegenerative effects [17]. Therefore, these circumstances have to be considered when targeting calpains for therapeutic purposes.

### 2.2. Autophagy

#### 2.2.1. Autophagy Basics

Cellular homeostasis is the result of constantly ongoing self-renewing processes that assure elimination of malfunctioning or nonfunctional components, from proteins to organelles. These highly conserved processes feed into recycling mechanisms that provide the cell with nutrients and metabolites. PTMs, typically ubiquitination, can mark proteins for destruction, if they are nonfunctioning, aggregating, or long-lived, eventually handing them over to the cell's major protein-degradation pathways: the ubiquitin-proteasome system (UPS) and the autophagy lysosome pathway (ALP) [56, 57].

Classically, UPS targets are tagged with K48-linked ubiquitin chains, recognized by the 19S regulatory cap of the 26S proteasome, unfolded, and then cleaved in the 20S proteolytic core, generating small peptides [58, 59]. ALP, the other degradational system, allows specific as well as bulk degradation under energy- and nutrient-deficient conditions. This system can be subdivided into three different mechanisms: chaperone-mediated autophagy (CMA), microautophagy, and (macro-)autophagy. All of them have the shuttling of cargo to the lysosome in common, where hydrolases break the content down to single amino acids. Whereas CMA relies on a specific KFERQ pentapeptide recognition sequence for the chaperone-mediated transport to the lysosomal transporters [60, 61], microautophagy is a rather unspecific engulfment of cytoplasmic content at the lysosomal membrane [62].

Macroautophagy is characterized by the* de novo* formation of double-membrane structures that are formed in the vicinity of the endoplasmic reticulum (ER), at nucleation sites, even though the origin of the membranes is still not entirely resolved [63]. Membrane structures are created that form the phagophore (isolation membrane) by a steady growth into the vesicular structure, engulfing cellular material from proteins up to organelles. The mature autophagosomes finally fuse with lysosomes to autolysosomes, where the cargo is degraded [64]. The genes responsible for this process (autophagy-related genes, ATGs) have been found by reverse genetics in* Saccharomyces cerevisiae* and a multitude of homologues were shown to be conserved throughout many species and in humans [65]. The sensory components of this degradation mechanism are the mechanistic target of rapamycin complex 1 (mTORC1) and AMP-activated protein kinase (AMPK), which integrate signals about nutritional cues and growth factors or the energetic status of the cell, respectively. This leads to the rapid adaptation of anabolic processes and to the release of amino acids, through recycling of cellular material by their differential regulation of the serine/threonine-protein kinase ULK1 [66, 67]. The formation of the autophagosome is classically initiated by the ULK1 (Atg1 in yeast) complex under nutrient deprivation. Beclin-1 is phosphorylated by ULK1 and VPS34 (Class III phosphatidylinositol 3-kinase (PI3K) in humans) is activated [68, 69]. In complex with VPS34, beclin-1 and ATG14 are involved in the nucleation of the phagophore and maturation of the autophagosome. The phagophore membranes are elongated via two ubiquitin-like systems (ATG12 and ATG8) by the reversible conjugation of several ATG gene products, which prime the growing ends for further protein interactions [70]. ATG5-conjugated ATG12 binds to ATG16L (E3-like protein) by E1-like (ATG7) and E2-like (ATG10) proteins. This complex at the extending phagophore allows the recruitment of the second ubiquitin-like system [71]. For this, ATG4-cleaved microtubule-associated protein 1 light chain 3 (LC3, Atg8 in yeast) is lipidated with phosphatidylethanolamine (PE). These modifications generate LC3-II [72], which is then incorporated into the double membrane. ATG7 and ATG3 function as E1-like and E2-like proteins, respectively, and LC3-II is conjugated to the ATG5/12/16L complex by ATG3, which drives the growth of the phagophore membrane [73, 74].

Autophagosomes can selectively engulf diverse forms of autophagic cargo, ranging from single proteins, over protein aggregates (aggrephagy), to whole organelles like mitochondria (mitophagy) and even proteasomes (proteaphagy) [75–78]. Cargo designated for degradation is detected by p62/SQSTM1, neighbor of BRCA1 gene 1 (NBR1), optineurin, Toll-interacting protein (TOLLIP), or other receptor proteins [78–80]. These receptors preferentially bind K63-polyubiquitin-tagged substrates and bring them in contact with the autophagosomes via a LC3-interacting region (LIR). The specific binding and the capacity of some proteins to act additionally as scaffolds for the recruitment of autophagic complexes ensure selective degradation [81].

Lastly, the mature autophagosomes fuse with lysosomes. This process requires several components, such as lysosome-associated membrane proteins (LAMPs) [82]. The degradation in the lysosome proceeds to the breakdown of proteinaceous cargo into single amino acids by cathepsins. Dysfunction of both degradative systems has been associated with neuronal aging and degeneration, bringing it into focus for therapeutic research [83].

#### 2.2.2. Autophagy in Health and Disease

In line with its essential role in cellular homeostasis, autophagy is involved in major disease classes like cardiovascular, infectious, and metabolic disorders as well as cancer [84]. It has been generally challenging to delineate the exact roles of autophagy in cell survival and cell death [85]. Whilst this mechanism can have cell protective functions in regard to genomic integrity [86] and autophagy induction is a common therapeutic strategy in cancer, inhibition of this pathway has also shown its applicability in tumor treatment. This is mainly because autophagy can represent an escape mechanism for tumor cells and may be responsible for the development of resistances [87].

In the healthy nervous system, autophagy relieves neurons of protein and organelle damage. Moreover, it plays an important role in developmental organization processes [88], ensuring axonal homeostasis [89] and sustaining the pool of neuronal stem cells [90]. Autophagy is most vital during the neonatal starvation period and thus ubiquitous deletion of ATG core proteins results in neonatal or embryonic lethality [91]. In brain injury by hypoxia or trauma, autophagy is a critical and protective factor in cell survival, underlining its important role in the survival of neurons [92, 93]. Conditional knockout of core autophagy genes leads to decreased life span and phenotypes resembling neurodegeneration [94, 95]. Successful aging is especially relevant in postmitotic cells such as neurons [96]. The accumulation of long-lived organelles and proteins, as well as the reduced ability of cells to cope with stress imposed by those, is believed to be a major cause for late onset neurodegenerative diseases. As many different pathomechanisms may lead to neurodegeneration, various disease-specific deregulations of the autophagic pathway have been suggested [81, 97].

In several neurodegenerative disorders of the brain, an accumulation of autophagosomes and autophagic markers has been observed [84, 98, 99]. Importantly, the mere finding of an increased number of autophagosomes gives no information on whether the autophagic flux is increased or the elimination of autophagosomes is just inhibited [100]. Observations in HD have shown that patient brain and lymphoblasts feature increased numbers of autophagosomes [101, 102]. This upregulation of autophagosome formation is caused by the sequestration and inactivation of mTOR by mutant huntingtin [103] and is accompanied by a defect in cargo loading [104]. Further, critical autophagy regulating genes, such as beclin-1 and Ras homolog enriched in striatum (Rhes), show reduced function and protein levels in HD brain [105–107]. Despite an already increased autophagy and functionally disturbed autophagosomes, genetic or pharmacological induction of autophagy has, however, been proven effective in different HD models [108, 109] and comparable results were obtained for other polyQ disorders as well as for AD and PD [110–113]. On the other hand, lysosomal cathepsins, which are responsible for the degradation of cargo proteins in autophagy, were associated with cleavage of mutant huntingtin in HD or APP in AD and, thus, formation of toxic fragments. In this regard, inhibition of these proteases led to beneficial effects on the molecular disease phenotype [114, 115]. Interestingly, not only have the disease-causing proteins in polyQ disorders been found to be degraded by autophagy, but also accumulated evidence suggests a direct role of proteins like huntingtin or MJD's ataxin-3 in autophagy regulation. Huntingtin itself represents a very special case since its structure is related to several ATG proteins. Consequently, it has been implicated in the induction of autophagy [116, 117]. Huntingtin, in its physiological function, is proposed to act as a scaffold, which recruits autophagy-initiating factors and adapter proteins [117, 118]. More recent studies have also found ataxin-3 to be a regulator of autophagy initiation. Wild-type ataxin-3 is a deubiquitinase that is thought to cleave polyubiquitin chains from beclin-1, thereby saving it from degradation and enabling autophagy [119]. By contrast, in MJD patient-derived fibroblasts, beclin-1 and autophagy levels were reduced, and beclin-1 overexpression rescued the deficit in autophagosome formation [120]. Interestingly, reduced beclin-1 levels are commonly detected in neurodegenerative disorders and aging brains, representing a limiting factor in autophagy induction and a driving factor in late onset proteinopathies [106, 121, 122].

Aside from polyQ disorders, different levels of autophagy deregulation have been reported for AD, PD, and ALS. An increased PI3K/AKT/mTOR signaling was shown in AD, as well as a defect in lysosomal clearance caused by A*β* [63, 121]. Autophagy induction by various means has been successfully tested in animal models of AD, and several substances have been evaluated in clinical trials [112, 123, 124]. All types of autophagy have been implicated in PD pathogenesis and macroautophagy, in particular, has been linked to mitochondrial dysfunction, due to ineffective mitophagy [125]. The PINK/parkin pathway, which is based on two proteins known to be causative for PD when mutated, regulates mitophagy and, therefore, controls mitochondrial number and quality. Moreover, the accumulation of *α*-synuclein has been found to interfere with mitochondrial turnover [126]. The genetic activation of autophagy by beclin-1 expression as well as pharmacological approaches were able to rescue disease phenotypes in PD animal models [113, 127, 128]. In ALS, the E478G mutation in the autophagy receptor optineurin leads to defective degradation of mitochondria [129]. Also, for the protein C9orf72, a regulatory function in autophagy induction has been proposed [130].

The general translation of findings on autophagy's role in cell and animal disease models to human patients and clinical applications poses a big challenge. Open questions remain about the exact dynamics of deregulation in autophagic processes in different neurodegenerative disorders, e.g., in terms of aging and tissue specificity. Furthermore, treatment approaches targeting mTOR and AMPK signaling pathways suffer from complications, such as pleiotropic effects or occurring toxicities. Thus, despite its compromised functionality in diseases, it is still unclear to which extent the autophagic clearance can be therapeutically exploited. In the pursuit of new targets for autophagy modulation, the calpain system could represent an approach to indirectly upregulate autophagy and thereby reestablish cell homeostasis.

### 2.3. Interplay between the Autophagy Pathway and the Calpain System

#### 2.3.1. Impact of Calpains on Autophagy

Due to their limited proteolytic activity and intrinsic substrate specificity, calpains are considered as modulator proteases, allowing them to regulate protein functions and, thereby, various cellular pathways. It is, therefore, obvious that calpains exert respective modulatory effects on autophagy. In many disease conditions and models, calpains were shown to negatively regulate autophagy, making enhanced calpain activation a conceivable contributory factor in the impaired activation of this degradation pathway.

**Figure 2:**
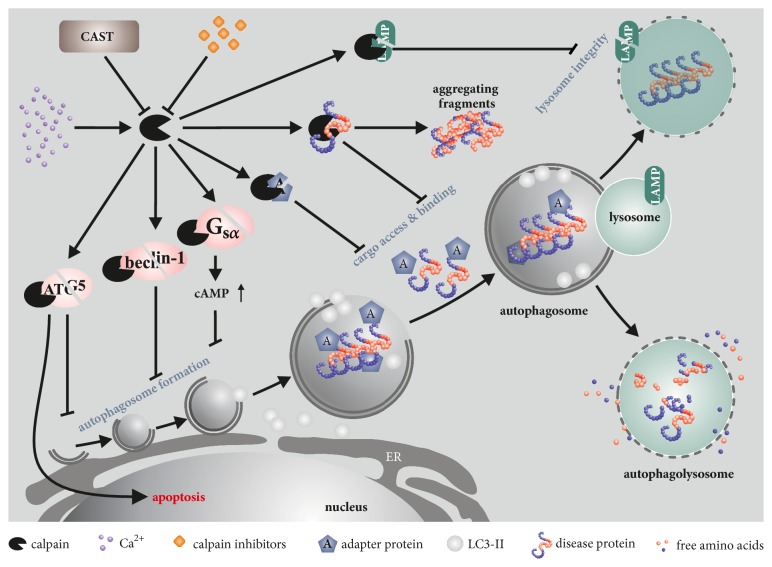
*Calpain targets in the autophagic machinery.* Calpains can impair protein clearance on different levels of the ALP. By cleaving signal transduction molecules, like G_s*α*_, or autophagic proteins, like beclin-1 and ATG5, calpains lead to a reduction of autophagy initiation and can, in the case of ATG5 cleavage, act as a switch from autophagy to apoptosis. Moreover, the cleavage of adapter proteins (optineurin, p62/SQSTM1), cargo (e.g., disease proteins), or lysosome-associated proteins (LAMPs) can change the dynamics of cargo degradation, thereby causing a defect in protein homeostasis. The inhibitory function of CAST reduces calpain activity and thereby leads to increased autophagy levels. Additionally, it prevents the cleavage of disease proteins into toxic or strongly aggregating fragments, rendering more soluble, full-length forms of the protein more accessible to autophagy.

Diverse studies have shown that the impact of calpains on autophagy occurs on multiple levels, as summarized in Figure 2. For instance, the *α*-subunit of heterotrimeric G proteins (G_s*α*_) appears to be a substrate for calpains. Cleavage leads to its activation, which in turn activates adenylyl cyclase. This results in an accumulation of cAMP, which then culminates in the inhibition of phagophore formation through activation of phospholipase C and, consequently, increased generation of inositol triphosphate (IP_3_) [131, 132]. Furthermore, ATG5 is cleaved and inactivated by calpains, leading to a disturbance of the ATG12-ATG5 complex formation and, as a consequence, of the expansion of the phagophore membrane [133, 134]. Interestingly, calpain-cleaved ATG5 was shown to translocate to mitochondria and induce apoptosis by blocking the antiapoptotic function of Bcl-xL. Thus, calpain cleavage of ATG5 constitutes a switch between autophagy and apoptosis [134]. Moreover, calpain overactivation as a result of anoxia-reoxygenation in cells or ischemia-reperfusion injuries* in vivo* demonstrated detrimental effects on autophagy via breakdown of beclin-1, ATG3, or ATG7, while calpain knockdown or overexpression of respective substrates counteracted the autophagic impairments [135–137]. Nearly all ATG proteins were shown to be cleaved by calpains* in vitro*, without characterizing, however, the biological relevance of their proteolysis [138]. Aside from proteins implicated in the proper formation of autophagosomes, calpains also target autophagy receptors, such as p62/SQSTM1 and optineurin, which may lead to a compromised cargo binding [138–140]. Autophagosome maturation might also be affected by calpains, as LAMP2 was shown to be cleaved by these proteases, leading to lysosomal permeabilization [141, 142]. Finally, due to their well-established role in microfilament dynamics, it was hypothesized that calpains may interfere with the dynamic changes of the cytoskeleton coupled to autophagosome formation [143]. Of note, the HD disease protein huntingtin, which is involved in autophagic processes, is a known calpain substrate, suggesting functional repercussions when proteolytically fragmented [43, 117, 118]. Interestingly, depletion of CSS1, whose knockout leads to early embryonic lethality in mice, induced lysosomal defects and blocked autophagy in cell-based experiments. The latter effect was attributed to the substantial calpain cleavage of Bif-1, which allows the scission of Golgi components and their targeting to nascent autophagosomes [144–146].

As neurodegenerative conditions, cancer, cardiovascular diseases, and diabetes have in common a reported deregulation of proteases and disturbances of the autophagic flux, further studies on the involvement of calpains in autophagy are of particular relevance. In the following sections, we will discuss the interplay between calpains and autophagy in a choice of those maladies.

#### 2.3.2. Interplay of Calpains and Autophagy in Diabetes, Ischemia and Cancer

Both calpain activation and deregulated autophagy are implicated in the molecular pathomechanisms of many common health conditions with unsolved or complex etiologies. Myriads of them feature an impaired Ca^2+^ homeostasis as a primary trigger for these disturbances.

In type 2 diabetes (T2D), amylin (or islet amyloid polypeptide, IAPP), a peptide hormone which is cosecreted with insulin in a ratio 1:100, was shown to accumulate in affected pancreatic *β* cells, forming amyloid deposits and, eventually, leading to cell death [147]. Autophagy has been suggested as a defending mechanism in *β* cells against the proteotoxicity of amylin, and a known dysfunction of the ALP in T2D may further contribute to detrimental effects [148, 149]. Interestingly, toxic amylin oligomers were shown to lead to intracellular membrane disruption, increased cytoplasmic Ca^2+^ concentrations, and, consequently, overactivation of the calpain system, specifically calpain-2, in cell models, mice, and pancreatic tissues from humans with T2D [150, 151]. This overactivation ultimately leads to critical autophagic dysfunctions [150].

Calpain overactivation is a general response during ischemia-reperfusion in many different tissues, when anaerobic metabolism decreases the active Ca^2+^ efflux and limits its reuptake by the ER, thereby producing Ca^2+^ overload in the cell. In the eye, heart, or liver, for instance, this overload leads to the deleterious overactivation of calpains, which then excessively cleave structural and functional proteins [152–154]. Following retinal ischemic injury* in vivo, *calpains were shown to fragment and inactivate beclin-1, resulting in the deregulation of autophagy [136]. A direct impact of calpains on autophagy remains unproven in the heart muscle; however, in fatty livers, calpain-2 inhibited autophagy by cleaving ATG3 and ATG7, thereby contributing to ischemia-reperfusion injuries [137]. In livers of obese mice, a baseline impairment of autophagy, due to ATG7 depletion, was associated with a dramatic increase in calpain-2 protein expression [155].

In cancer cells, calpains are often mediating the switch between protective autophagy and desired apoptosis (e.g., via ATG5). Inhibiting their activity may be disadvantageous in anticancer treatments, whereas activating them may be beneficial [156, 157]. Investigations on human metastatic melanoma cells treated with cisplatin revealed that this chemotherapeutic induces calpain activation and inhibits basal autophagy, while autophagy activation by calpain inhibition acts as a prosurvival response [158, 159]. A nonclassical calpain demonstrated a different effect regarding the control of the autophagy system in sarcoma cells. Knockdown of calpain-6, which is strongly upregulated in cells with tumor-initiating and metastatic capacities, suppressed autophagy as well as hypoxia-dependent prevention of senescence entry [160, 161]. The Kaposi sarcoma-associated herpesvirus inhibits autophagy and impairs monocyte differentiation into dendritic cells as an immune evasion strategy, by reducing CAST expression and consequently leading to decreased ATG5 levels [162].

#### 2.3.3. Linking Calpain Activation and Autophagy in Neurodegeneration

The neurodegenerative disorders AD, ALS, HD, MJD, and PD exhibit an overactivation of calpains and disturbances of the autophagic pathway [1, 8, 81]. Considering the known implications of calpains in autophagy, a link between both pathways is also apparent in neurodegeneration.

In HD, calpains have been early identified as a disease modifier, being overactivated in the disease context and leading to cleavage of polyQ-expanded huntingtin [43, 49]. Likewise, a deregulation of autophagy was shown for HD, which is further emphasized by wild-type huntingtin's physiological involvement in this pathway [117, 118, 163]. A direct connection between calpain overactivation and autophagy deregulation has yet not been made for HD; however, the knockdown of a calpain homologue in an HD* Drosophila* model and CAST overexpression in HD mice reduced polyQ toxicity of an N-terminal huntingtin fragment and improved behavioral signs, by activating autophagy [164]. This upregulation of the autophagic pathway was attributed to cleavage inhibition of the calpain substrate G_s*α*_, as shown earlier in cell and zebrafish models of HD [131]. Respective effects also cannot be ruled out as a contributing factor in two of our preclinical studies, where we treated two HD animal models with the experimental drug olesoxime, thereby not only reducing calpain overactivation, huntingtin fragmentation, and aggregate formation, but also ameliorating the behavioral phenotype [47, 165]. In an MJD zebrafish model, calpain inhibition reduced polyQ-expanded ataxin-3 levels in an autophagy-dependent manner [166]. In a conditional *α*-synuclein-expressing mouse model of PD, the environmental neurotoxin paraquat was shown to activate calpains, leading to inhibition of autolysosomal clearance and, thereby, accumulation of both calpain-cleaved and insoluble *α*-synuclein species [167].

Calpains were, furthermore, suggested to act as a switch between two modes of cell death in hippocampal neural stem cells, as low calpain activity triggered by insulin deprivation resulted in a preference for autophagic cell death over apoptosis [168]. In cortical neurons, autophagy was shown to fail preventing glucose deprivation/reintroduction-induced neuronal death due to lysosomal permeabilization, which resulted from a calpain-mediated LAMP2 cleavage [141].

### 2.4. Activating Autophagy via Calpain Inhibition as a Therapeutic Approach

#### 2.4.1. Genetic Approaches for Calpain Inhibition to Stimulate Autophagy

In the previous paragraphs we have highlighted the relevance of calpains and autophagy in various human medical conditions, as well as the interplay of both proteolytic machineries. The fact that calpains have a direct regulatory impact on the autophagic system suggests the assumption that exclusively targeting these proteases may target both their deregulation and the autophagic dysfunction. Not having reached clinical applicability yet, multiple cell-based or* in vivo* disease models have delivered general proofs of concept using genetic or pharmacological approaches.

Typical genetic strategies comprise the overexpression of the endogenous calpain inhibitor CAST, or the knockdown and knockout of calpain isoforms as well as of CSS1. In a human IAPP transgenic mouse model of T2D, overexpression of CAST was shown to be protective against the loss and dysfunction of pancreatic *β* cells and preventing diabetes onset by restoring the vital ALP [150]. Moreover, the intravitreal injection of siRNA directed against CSS1 reduced calpain activation and beclin-1 cleavage in an* in vivo* model for retinal ischemic injuries [136]. In a mouse model for bone sarcoma, knockdown of the nonclassical calpain-6 blocked tumor development, and overexpression of this protease in an osteosarcoma cell line increased autophagic flux, which could rather favor tumorigenesis [161].

In models of neurodegenerative disorders, CAST overexpression and calpain knockdown were protective against toxicity of the respective disease proteins. RNAi-mediated knockdown of the calpain homologue* CalpA *in* Drosophila* models of HD and tauopathy ameliorated the disease-related phenotypes in an autophagy-dependent manner [164]. HD mice transgenic for CAST showed an activation of autophagy, leading to reduced mutant huntingtin protein and aggregate levels, attenuating disease symptoms, such as tremor and motor phenotype [164]. In line with these findings, our group showed that HD mice with ablated CAST expression presented, in addition to calpain overactivation and the consequent increase of huntingtin cleavage and aggregation, disturbances in autophagy [169]. Analogously, positive effects of CAST overexpression as well as negative consequences of an CAST knockout on disease protein toxicity were shown in further cell and mouse models of neurodegenerative disorders, such as ALS, PD, and SCA3, without, however, linking it directly to autophagy [44, 45, 48, 50, 52–54, 170].

Excessively calpain inhibition can be, nevertheless, detrimental, as the inhibition of calpain activity by CAST overexpression in mouse hearts resulted in a progressive cardiomyopathy characterized by accumulation of protein aggregates, formation of autophagosomes, and disruption of sarcomere integrity [171]. Moreover, in CAST transgenic mice, postinfarct scar healing was impaired, leading to an increased mortality [172].

#### 2.4.2. Pharmacological Approaches for Calpain Inhibition to Stimulate Autophagy

Pharmacological inhibition of calpains for stimulating autophagy can be achieved in two modes: first, by targeting calpains directly with specific inhibitors and, second, by aiming at calpain-activating mechanisms (i.e., primary elements of the cellular Ca^2+^ homeostasis). Accordingly, studies in different fields of clinical investigations have tested these respective approaches.

In a model for retinal ischemic injuries, administration of calpain inhibitors MDL 28170 and SJA6017 prevented calpain overactivation and cleavage of beclin-1 [136]. Pharmacological inhibition of calpain-2, not calpain-1, suppressed anoxia-reoxygenation-induced loss of autophagy proteins beclin-1 and ATG7, avoiding the onset of mitochondrial permeability transition and decreasing cell death after reoxygenation in rat hepatocytes [135]. Similar results were achieved by preventing a Ca^2+^ overload in mouse livers after ischemia-reperfusion, using the anticonvulsant/antiepileptic drug carbamazepine, which suppressed the calpain-mediated autophagic flux impairment and likewise prevented the loss of beclin-1 and ATG7 [173]. Furthermore, inhibition of calpain-2 in steatotic livers restored autophagic flux both in livers from obese rats, after ischemia-reperfusion, and in free fatty acid-treated hepatocytes [174]. In obese mice, administration of calpain inhibitors MDL 28170 and PD150606 resulted in rescued ATG7 level, eventuating in lower ER stress, enhanced hepatic insulin action, and systemic glucose tolerance [155].

In the field of polyQ diseases, diverse approved compounds have been rising as a promising reliever, through calpain inhibition, for those disorders. In PC12 and neuroblastoma cells expressing an exon 1 fragment of mutant huntingtin, inhibition of L-type Ca^2+^ ion channels (e.g., with verapamil) enhanced the autophagic clearance of the soluble and aggregated disease protein in a calpain-inhibiting manner. These effects could also be observed by direct inhibition of calpains using calpeptin [131]. In line with this, treatment of an MJD zebrafish model with calpeptin resulted in lowered mutant ataxin-3 expression by increased autophagy, which furthermore ameliorated the model's motor phenotype [166]. With regard to its significant effects on calpain activation and mutant huntingtin aggregation in two animal models of HD, the voltage-dependent anion channel-targeting experimental drug olesoxime may most likely exert positive effects on autophagy. However, respective evidence still needs to be provided [47, 165].

## 3. Conclusions

As presented in this review, many* in vivo* and* in vitro* studies have furnished evidence for the crosstalk between calpains and autophagy, two important proteolytic cell machineries. The interaction is grounded on calpains' functional role as* modulator proteases*, which cleave and modify the activity of multiple substrate proteins, in this context, involved in the autophagy pathway. Thereby, calpains regulate autophagy by influencing autophagosome formation, substrate recognition, and cargo degradation.

This direct mechanistic impact highlights calpains as a point of vantage for therapeutically targeting autophagy. Especially in neurodegenerative diseases, but also other medical conditions such as diabetes or ischemia, where calpains are known to be overactivated, genetic or pharmacological strategies to inhibit these proteases can attenuate concomitant autophagic disturbances. Still, a further and broader dissection of the interplay is necessary, which may cover its potential involvement in other diseases and additional underinvestigated processes along the autophagy pathway. Notwithstanding the known detrimental effects of an exhaustive calpain inhibition or an excessive autophagy activation, the general role of calpains in autophagy regulation may present downstream advantages, either by increasing cell viability such as in diabetes and neurodegeneration, or by facilitating apoptotic cell death upon chemotherapy in cancer.

For this reason, a broad discernment on both proteolytic machineries and their intersections may be useful for understanding deregulation of calpains and autophagy in neurodegenerative diseases and beyond, thereby contributing to the development of additional treatment strategies for a multitude of medical conditions.
